# Electroacupuncture could balance the gut microbiota and improve the learning and memory abilities of Alzheimer’s disease animal model

**DOI:** 10.1371/journal.pone.0259530

**Published:** 2021-11-08

**Authors:** Jing Jiang, Hao Liu, Zidong Wang, Huiling Tian, Shun Wang, Jiayi Yang, Jingyu Ren

**Affiliations:** Beijing University of Chinese Medicine, Beijing, China; Shanghai Jiao Tong University, CHINA

## Abstract

Alzheimer’s disease (AD), as one of most common dementia, mainly affects older people from the worldwide. In this study, we intended to explore the possible mechanism of improving cognitive function and protecting the neuron effect by electroacupuncture. Method: We applied senescence-accelerated mouse prone 8 (SAMP8) mice as AD animal model, used Morris water maze, HE staining, 16S rDNA amplicon sequencing of gut microbiota and ELISA to demonstrate our hypothesis. Results: electroacupuncture improved the learning and memory abilities in SAMP8 mice (P<0.05) and could protect the frontal lobe cortex and hippocampus of SAMP8 mice; electroacupuncture significantly decreased the expression of IL-1β (P<0.01), IL-6 (P<0.01) and TNF-α (P<0.01 in hippocampus, P<0.05 in serum) in serum and hippocampus; electroacupuncture balanced the quantity and composition of gut microbiome, especially of the relative abundance in *Delta-proteobacteria* (P<0.05) and *Epsilon-proteobacteria* (P<0.05). Conclusion: electroacupuncture treatment could inhibit the peripheral and central nerve system inflammatory response by balancing the gut microbiota.

## 1 Introduction

Alzheimer’s disease (AD), as one of most common dementia, which mainly affects elderly people, gains more and more awareness from the worldwide. According to the survey of Alzheimer’s disease International (ADI), there are over 9.9 million new cases of dementia each year worldwide, implying one new case every 3.2 seconds [1]. Since researchers first recognized this disease, they never stopped to find the pathological process and explore the effective therapies. Nowadays, not only the senile plaques (SPs) formed by deposition of amyloid-β protein [2] and neurofibrillary tangles (NFTs) formed by hyperphosphorylation of tau protein [3] were viewed as the characteristics of AD, but also the neuroinflammatory reaction participated by glial cells were viewed as the key pathological changes of AD [4]. Unfortunately, there are still no effectiveness therapies, which could reverse or terminate the process of the disease [5].

In recent years, increasing evidence showed that the dynamic changes in the gut microbiota could alter brain physiology, cognition and the process of some diseases, such as pain [6], depression [7], anxiety [8], stroke [9] and Alzheimer’s disease [10, 11]. With further research, more evidence proved that the gut microbiota could regulate and influence cognitive dysfunction as well as the process of neurodegeneration and cerebrovascular diseases by regulating the composition and structure of the microbiota [12]. Moreover, significant quantitative and qualitative changes of gut microbiome were reported in patients with Alzheimer’s disease [13, 14]. The possible reasons for these changes might be associated with external lifestyle aspect, such as diet [15], sleep deprivation, circadian rhythm disturbance, chronic noise, and sedentary behavior, which were also considered as important risk factors for the development of sporadic AD [16]. More and more evidence proved that the changes of metabolites produced by gut microbiome significantly influenced intestinal barrier function and neuroinflammation process in brain, which were also considered as key pathological process in AD [17]. Therefore, finding more and further evidence between gut microbiota and the process of AD became one of research focuses in pathology of AD.

Since there were no curable therapies for AD, improving the clinical symptoms and effectively controlling the risk factors were the main strategies for treatment of this disease [18]. Electroacupuncture therapy, based on the basic theories of Chinese medicine and widely used in clinic not only in China but also worldwide, has been proved the improving effects of cognition for AD patients in clinics [19–22]. Therefore, more researchers are interested in the mechanism of electroacupuncture, and also want to find some valuable clues for prevention and treatment of AD.

Our research team have been concerned to find more evidence of the mechanism of electroacupuncture intervention AD. From our former studies, we proved electroacupuncture therapy could regulate the metabolism level of glucose and cerebral blood flow in the brain of AD animal models [23, 24], and also could inhibit the neuroinflammation via regulating the activation of microglia [25–27]. In this study, we intended to explore whether electroacupuncture could take part in the regulation of gut microbiota and neuroinflammation of AD animal model, and the possible mechanism of these.

## 2 Materials and methods

### 2.1 Animals and ethics statement

Eight-month-old senescence-accelerated mouse prone 8 (SAMP8) mice and senescence-accelerated mouse resistant 1 (SAMR1) mice weighing 30.0 ± 2.0 g were purchased from the Experimental Animal Center of First Teaching Hospital of the Tianjin University of Traditional Chinese Medicine (Animal Lot: SCXK (Jing) 2014–0003). The animals were housed at a controlled temperature (24 ± 2°C) and under a 12-h dark/light cycle, with sterile drinking water and a standard pellet diet available ad libitum. All mice were acclimatized to the environment for 5 days prior to experimentation. All procedures were complied with the ARRIVE guidelines were performed according to the guidelines of the National Institutes for Animal Research (ID: BUCM-4-2018111701-4045).

### 2.2 Grouping and intervention

8 SAMR1 male mice were used as the normal control (Control group) and 24 SAMP8 male mice were randomly divided into three groups (*n* = 8 per group): AD model control group (AD group), drug group (Drug group), and Electroacupuncture group (EA group). In the Drug group, donepezil hydrochloride tablets (Eisai China Inc., H20050978) were dissolved in distilled water and delivered to mice by oral gavage at a dose of 0.65 μg/g. In the EA group, mice were immobilized in mouse bags. *Baihui (GV20)* and *Yintang (GV29)* were chose for electroacupuncture for 15min per day, with transverse puncturing at a depth of 4–5 mm using disposable sterile acupuncture needles (0.25 mm × 13 mm) (*Beijing Zhongyan Taihe Medicine Company*). The needles were taped and connected to the HANS- LH202 electroacupuncture device (*Peking University Institute of Science Nerve and Beijing Hua Wei Industrial Development Company*, *Beijing*, *China*), with the sparse wave at 2 Hz, 2 V, and 0.1 mA. The mice in the Control group, AD group, and Drug group received the same 15-min restriction as the EA group.

### 2.3 Morris water maze

After 15-day intervention, mice from each group were evaluated in the Morris water maze. The Morris water maze consisted of a circular tank (diameter, 120 cm; height, 50 cm) filled with opaque water, rendered with milk powder to a depth of 30 cm. A video camera (TOTA-450d, Japan) fixed to the ceiling and connected to a video recorder with an automated tracking system (China Daheng Group, China) was used to automatically collect data. A removable platform (diameter, 9.5 cm; height, 30 cm) was placed inside the pool (at quadrant III). The pool area was conceptually divided into four quadrants (I, II, III, and IV) of equal size. Visual cues of different shapes were placed on the tank wall of each quadrant in plain sight of the mice. Hidden platform trial was used for testing the spatial learning ability of the mice. Probe trial was used for testing the spatial memory ability of the mice.

### 2.4 DNA extraction and library construction

After the Morris water maze test, the feces of each mouse were collected and frozen to ultra-low temperature refrigerator until test. Total genomic DNA was extracted using DNA Extraction Kit following the manufacturer’s instructions. Concentration of DNA was verified with NanoDrop and agarose gel. The genome DNA was used as template for PCR amplification with the barcoded primers and Tks Gflex DNA Polymerase (Takara). For bacterial diversity analysis, V3-V4 variable regions of 16S rRNA genes was amplified with universal primers 343 F and 798 R. (343 F: 5’- TACGGRAGGCAGCAG -3’; 798 R: 5’- AGGGTATCTAATCCT-3’). Amplicon quality was visualized using gel electrophoresis, purified with AMPure XP beads (Agencourt), and amplified for another round of PCR. After purified with the AMPure XP beads again, the final amplicon was quantified using Qubit dsDNA assay kit. Equal amounts of purified amplicon were pooled for subsequent sequencing. The libraries were sequenced on Illumina MiSeq platform (Illumina, San Diego, CA, USA) to generate 2 × 250-bp pair-end sequencing reads according to the standard protocol provided by Illumina.

### 2.5 Sequence analysis

PE reads were spliced through the overlap by using FLASH v1.2.11 software, then the original Tags data (Raw Tags) was obtained. High quality Tags were generated by filtering the raw tags in Trimmomatic v0.35 software. We used UCHIME v2.4.2 software to identify and remove chimeras Sequence, and to get the final effective data (Valid Tags). Operational taxonomic unit (OTU) was clustered and taxonomic annotation were performed in QIIME (version 1.8.0). The abundance of species at different levels (phylum, class, order, family, genus, and species) of classification was also by performing the QIIME software (version 1.8.0). linear discriminant analysis (LDA) coupled with effect size measurements (LEfSe) was used for screening significantly different biomarkers. Indicator analysis using R software package, through the calculation of OTUs in each group, and then statistical analysis of indicators species of each group.

### 2.6 H&E staining

To observe the neuron in frontal lobe cortex and hippocampus of each group, after the Morris water maze tests, 3 mice randomly chosen from each group were anesthetized, and their brains were removed and subjected to H&E staining. Tissues were dehydrated, paraffin-embedded, and sliced (thickness, 10 mm/slice), followed by dewaxing 3 times in xylene for 5 min each time and then placing them in anhydrous ethanol for 5 min, followed by 90% ethanol, 70% ethanol, and distilled water for 2 min. And then stained with hematoxylin and eosin (H&E). After staining, sections were dehydrated through increasing concentrations of ethanol and xylene. Dentate gyrus of hippocampus in each Specimen was viewed under an Olympus light microscope (Olympus Corporation, magnification, ×40).

### 2.7 ELISA quantification of proinflammation factors

Other mice of each group were sacrificed under anesthesia for their hippocampus and blood. ELISA detection was performed according to conventional ELISA method, the concentrations of proinflammatory factors IL-1β (*Raybiotech*, ELM-IL1b-CL-1; *proteintech*, KE10003), IL-6 (*Raybiotech*, ELM-IL6-CL-1; *proteintech;*KE10007) and TNF-α (*Raybiotech*, ELM-TNFa-CL-1; *proteintech*, KE10002) in hippocampus and serum (dilution concentration1:5) were detected respectively, and the specific steps were carried out strictly according to the instructions of the kit.

### 2.8 Statistical analysis

Statistical analysis was performed with software SPSS, version 25.0 (SPSS, Inc., Chicago, IL, USA), and data were expressed as mean±standard deviation (x±s). One-way ANOVA was used after the test of normal distribution and homogeneity of variance, and LSD method was used for pairwise comparisons. If there was a non-normal distribution or heterogeneity of variance for the data, a non-parametric test would be used. Statistical significance was set to p< 0.05, while highly statistical significance was set to p< 0.01.

## 3 Results

### 3.1 Electro-acupuncture could improve the learning and memory abilities in SAMP8 mice

**Fig 1:**
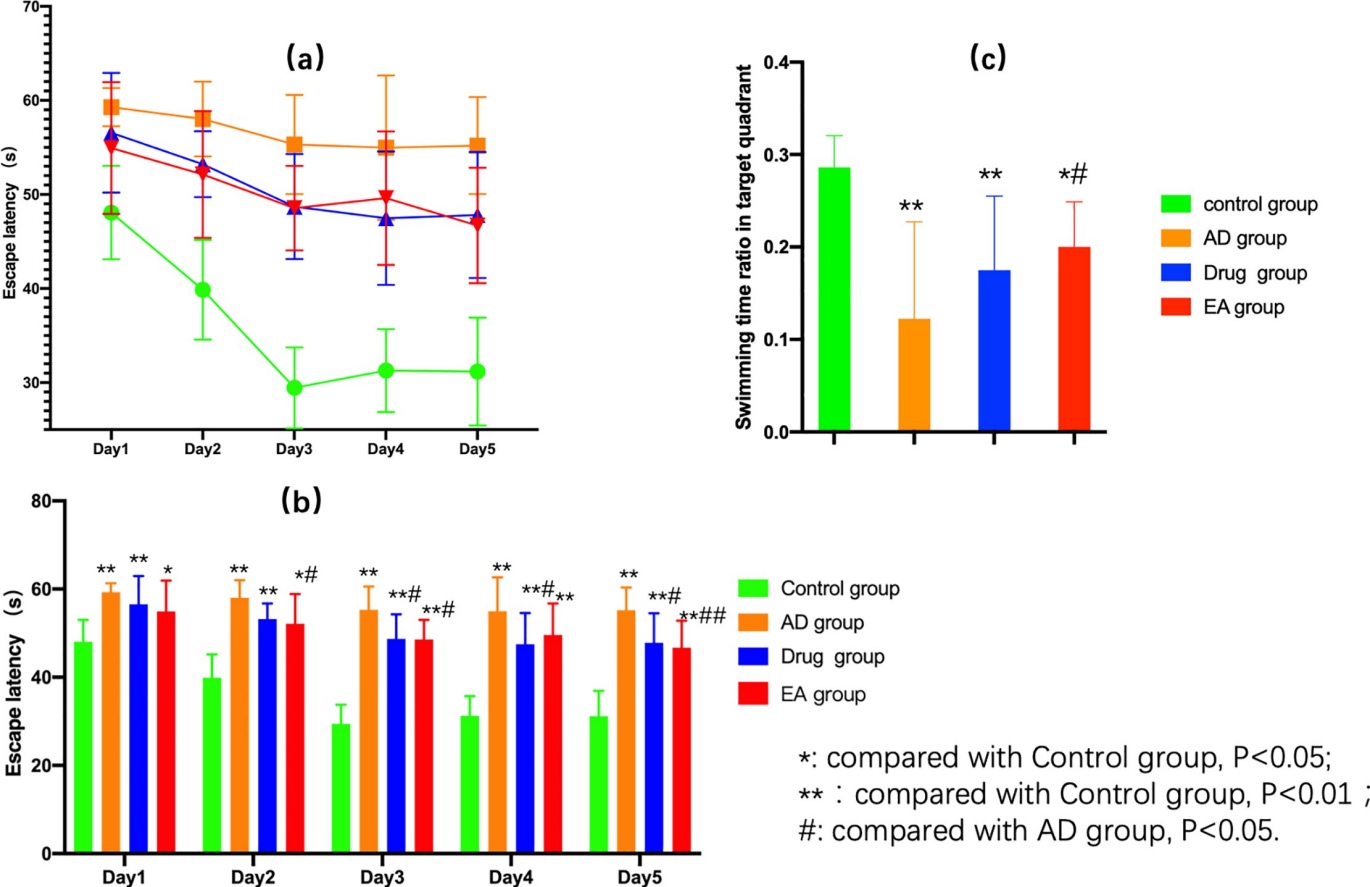
Results of Morris water maze test. (a, the tends of escape time in each group during the five training days of hidden platform test; b, comparing the escape time of each group during the five training days of hidden platform test; c, the ratio of swimming time in the target quadrant and total swimming time of each group in the probe test.).

The Morris water maze test was used to measure the spatial learning and memory abilities of the animal models. The first day to the fifth day was hidden platform test, which was designed to measure the spatial learning ability of the mice. From these we found the escape time in hidden platform test of each group shortened according to the training days accumulated (Fig 1A). Moreover, the shortening of escape latency was the most obvious in control group (compared with other three group, from the first day to the fifth day, P<0.05); compared with AD group, the escape time was significantly shortened from the third day to the fifth day (P<0.05); on the second day and the fourth day, the escape time of EA group was significantly shorter than AD group (P<0.05), especially on the last day (P<0.01) (Fig 1B). After the hidden platform was removed, the swimming time in the target quadrant and the total swimming time were recorded in the probe test. As showed in Fig 1C, analysis by one-wag ANOVA displayed the distinguished difference of the swimming time radio in target quadrant. The radio in control group was the highest (compared with other three group, P<0.05) and the EA group was higher than AD group (P<0.05). These results demonstrated electroacupuncture therapy could improve the spatial learning and memory abilities of SAMP8 mice, and at some extent, electroacupuncture therapy performed better in improving the memory abilities of SAMP8 mice. What’s more, we also found the results of EA group on the fourth day was a little bit higher than the third day, we assumed this might result from the training fatigue. The fifth day’s results affirmed this at some extent because of the results were better than the third and fourth day.

### 3.2 Electro-acupuncture could protect the neuron in the frontal lobe cortex and hippocampus of SAMP8 mice

**Fig 2:**
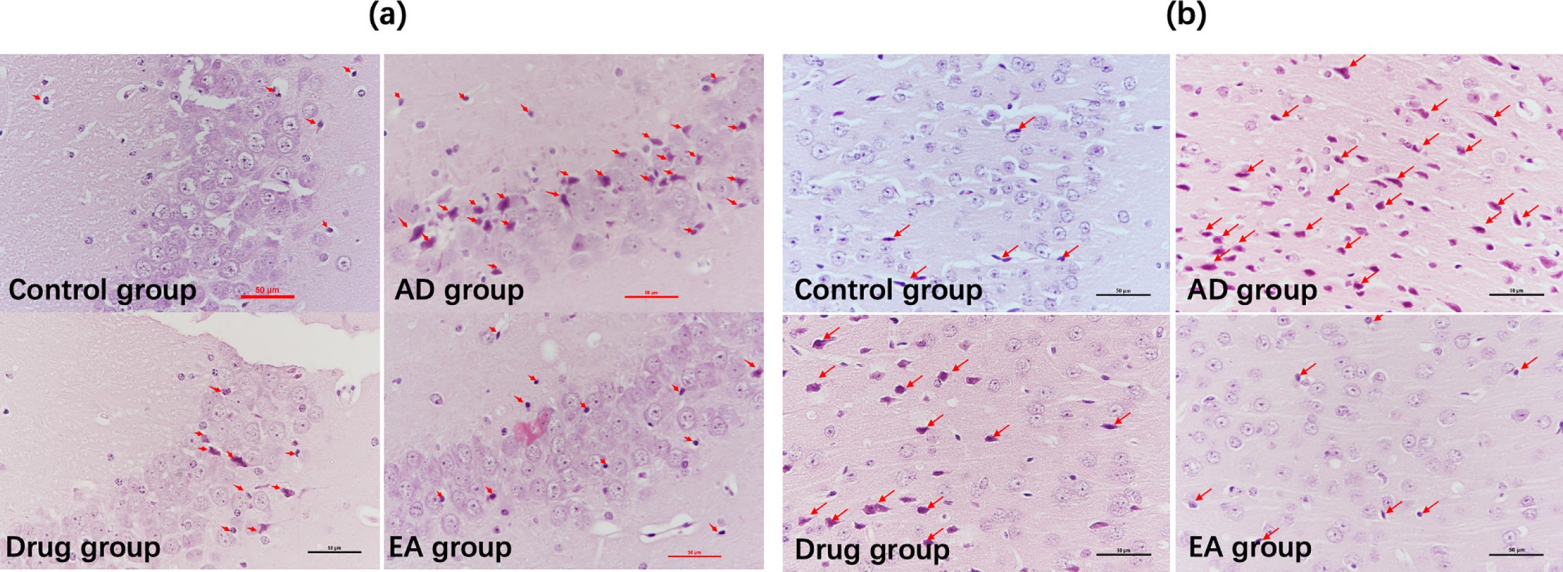
Results of HE staining. (a, the HE staining of each group at hippocampus, *400; b, HE staining of each group at frontal lobe cortex, *400.).

Fig 2 showed the CA3 region in hippocampus (Fig 2A) and frontal lobe cortex (Fig 2B) of the HE staining in each group. Mice of control group demonstrated clear-dyed neurons aligned in neat rows, with round nuclei and distinct kernels in the CA3 region in hippocampus and frontal lobe cortex. Conversely, neurons tended to be scattered and irregular, with indistinct kernels and nuclear pyknosis in these regions of AD group. Subjectively, compared with AD group, neurons were more neatly arranged in rows and clearer in structure with less nuclear condensation in Drug and EA groups. These demonstrated electroacupuncture could protect the neuron in the CA3 region of hippocampus and frontal lobe cortex at some extent.

### 3.3 SAMP8 mice showed a remarkably different composition of gut microbiota compared to SAMR1 mice

**Fig 3:**
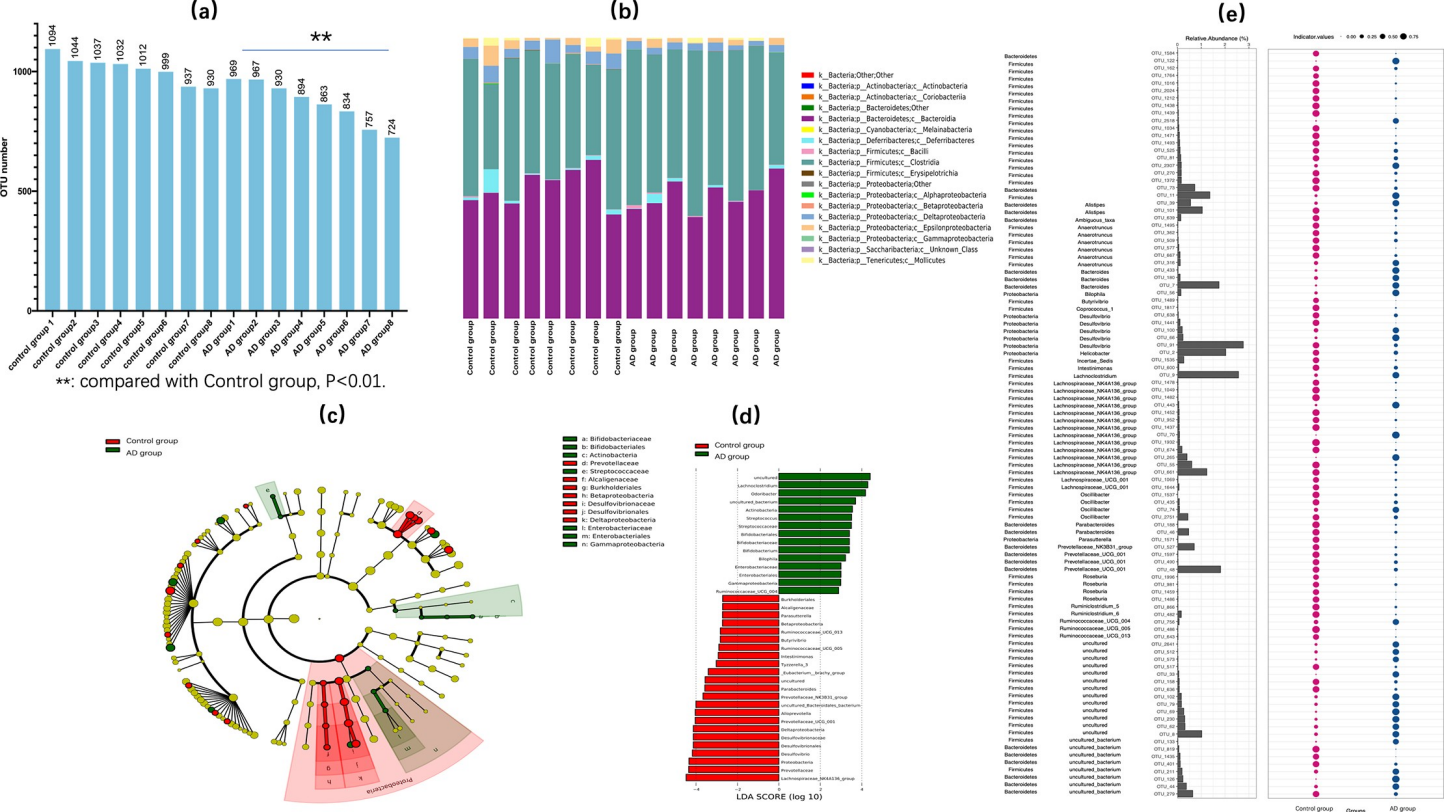
Differences of SAMP8 and SAMR1 mice in Gut Microbiota. (a, comparing the OTUs numbers of each sample in control group and AD group; b, gut microbiota community structure of control group and AD group; c-d, linear discriminant analysis (LDA) coupled with effect size measurements, different species of control group and AD group; e, indicator analysis of indicator species in control group and AD group.).

To collect more evidence to assess the potential correlations between gut microbiota and AD, we sequenced the fecal samples of each group. The number of clean tags ranged from 41849 to 51693. After removing the chimeras, the valid tags, which was the final data for analysis, was obtained. The data size was 37158 to 45742. The operational taxonomic units (OTUs) in each sample were 724 to 1094. We found the OTUs of SAMP8 mice were significantly lower (P<0.01) than that of SAMR1 mice (Fig 3A), indicating a lower gut microbiota abundance in SAMP8 mice. Community structure, or "biological community", referred to all organisms, that had direct or indirect relationship with each other in the biological environment of a community. In the microbial community, various groups interacted with each other and could coexist in a regular manner. At the same time, they had their own obvious nutritional and metabolic types. Fig 3B showed the community structure of each sample in control group and AD group. We used the LEfSe analysis to reveal the composition of different species in SAMP8 mice and SAMR1 mice in biological communities. The red nodes indicated the species with relatively high abundance in control group, green nodes indicated the species with relatively high abundance in AD group, and the yellow nodes indicated the species with no significant difference between the two groups. The diameter of the nodes was directly proportional to the relative abundance. The nodes in each layer represent phylum / class / order / family / genus from the inside to the outside (Fig 3C). In the score chart of different species, the red bars indicated higher relative abundance in control group, and the green bar indicated higher relative abundance in AD group (Fig 3D). Indicator analysis using R software package, through the calculation of OTUs in each group, and then statistical analysis of indicators species between control group and AD group (P < 0.05). The indicator analysis mainly referred to the indicator species in each group, which means the species that could have a greater impact on the growth environment in a certain area (Fig 3E). From the above results, we found the SAMP8 mice had significantly differences between SAMR1 mice in the number of OTUs, the structure of communities, the Composition of different species in biological community and the indicator species.

### 3.4 Electro-acupuncture could modulate the imbalance of gut microbiota in SAMP8 mice and inhibit the proinflammation factors in serum and hippocampus of SAMP8 mice

In this study, we used the donepezil, which was widely used in clinic for AD, as the medicine control, to evaluate the anti-dementia effect and regulating effect on gut microbiota. We collected fecal samples of each mouse from Control group, AD group, Drug group and EA group.

**Fig 4:**
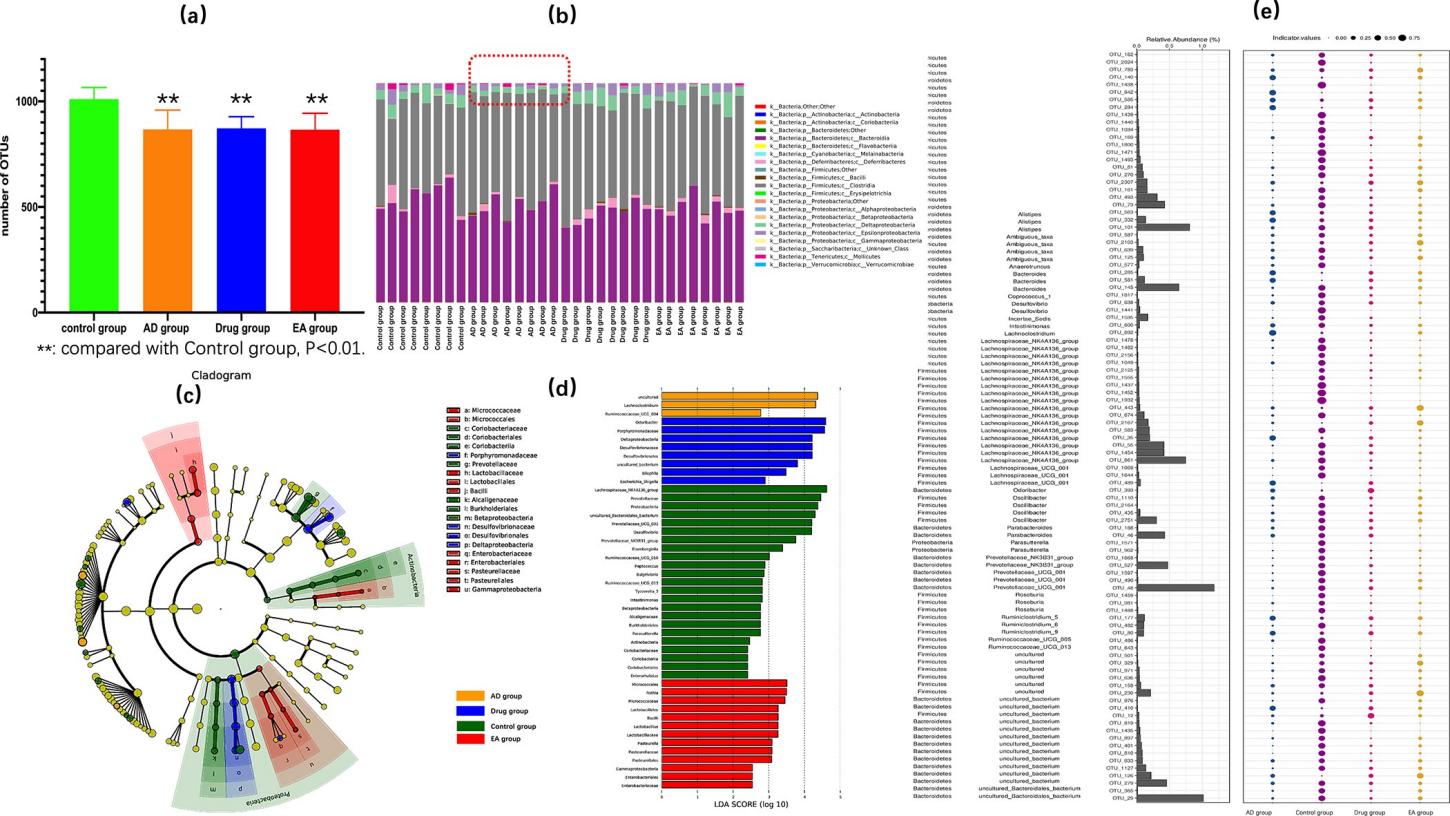
(a, comparing the OTUs numbers of each group; b, gut microbiota community structure of each group; c-d, linear discriminant analysis (LDA) coupled with effect size measurements, different species of each group; e, indicator analysis of indicator species in each group.).

Frist, we compared the number of OTUs of each group. We found only the control group had great differences between AD group (P<0.01); drug group and EA group had no significant differences with AD group (Fig 4A). Then we analysis the community structure of each group. From the Fig 4B, we could see a clear difference between AD group and other three groups in the structure of gut microbiota community (labeled with the red line). We used the LEfSe analysis to reveal the composition of different species in the four groups. As we could see in the Fig 4C & 4D, the species with high relative abundance in each group were different, from which we also could found EA group and drug group have more species in high relative abundance than AD group. Based on the OTUs, the indicator analysis showed the indicated species of each group (Fig 4E). Although there was significant difference between control group and other three groups, we could also find some differences among AD group, drug group and EA group in some species.

**Fig 5:**
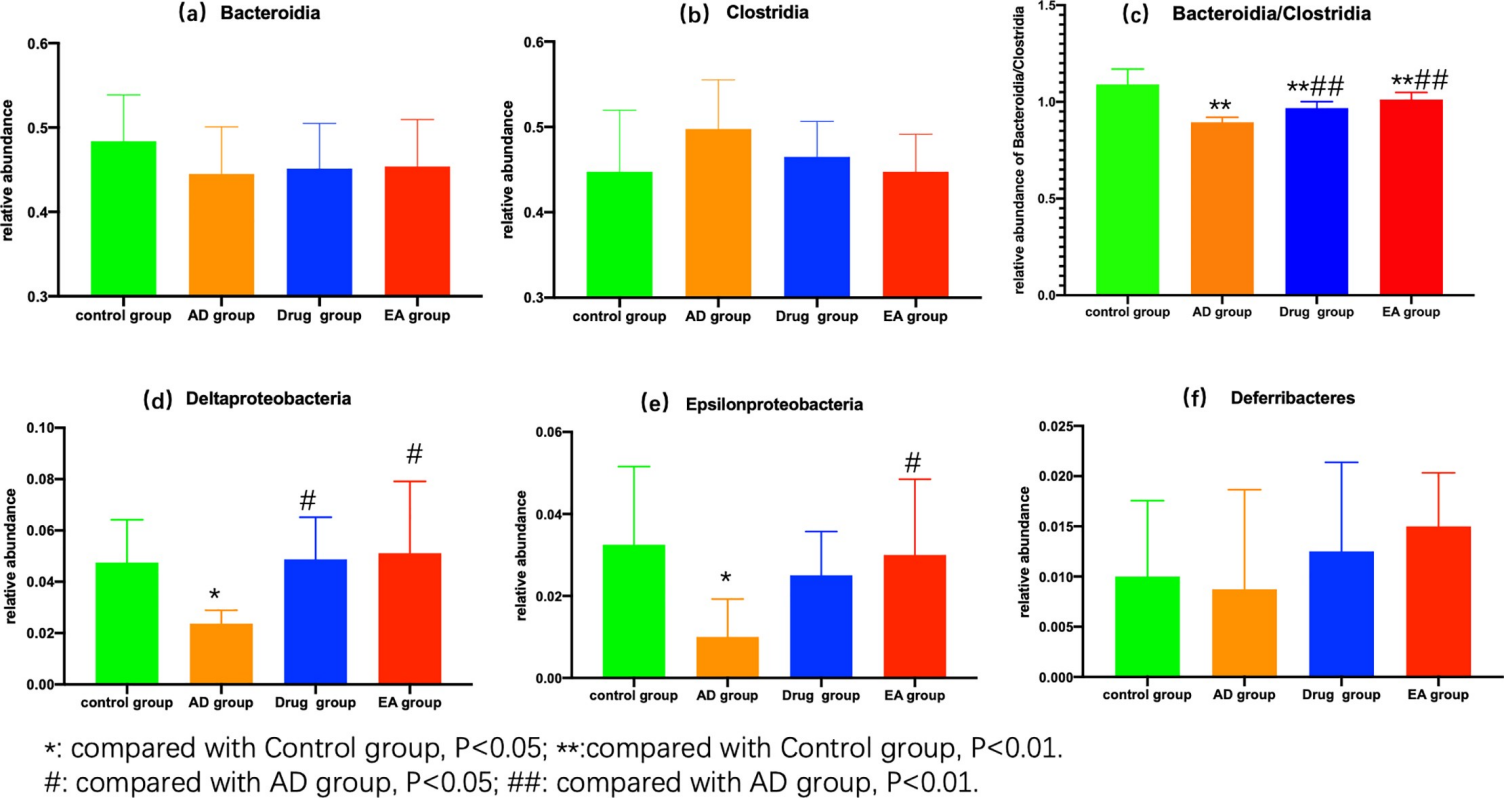
(a, comparation the relative abundance of *Bacteroidia* in each group; b, comparation the relative abundance of *Clostridia* in each group; c, comparation the ratio of *Bacteroidia* and *Clostridia* in relative abundance of each group; d, comparation the relative abundance of *Delta-proteobacteria* in each group; e, comparation the relative abundance of *Epsilon-proteobacteria* in each group; f, comparation the relative abundance of *Deferribacteres* in each group.).

Second, we analyzed the dominant bacteria of each group. According to the taxonomic category, we extracted the top-15 classes of each group for analysis. As seeing from the community structure (Fig 4B), *Bacteroidia* and *Clostridia* were the dominant bacteria of each group, and there was no significant difference among the four groups in relative abundance (Fig 5A and 5B). Interestingly, when we analysis the ratio of *Bacteroidia* and *Clostridia* of each group, there were significant statistical differences in Fig 5C. Compared to control group, the other three groups were lower in the ratio of *Bacteroidia* and *Clostridia* (P<0.01). Moreover, compared with AD group, drug group and EA group were higher in this ratio (P<0.01). Besides this, we also found there were significant differences in the relative abundance of *Delta-proteobacteria* and *Epsilon-proteobacteria* (Fig 5D and 5E). Compared with control group, AD group was lower in relative abundance of both *Delta-proteobacteria* and *Epsilon-proteobacteria* (P<0.05). Compared with AD group, the relative abundance of drug group and EA group were both higher in *Delta-proteobacteria* (P<0.05), and EA group was higher in *Epsilon-proteobacteria* (P<0.05). Others of top-15 classes in each group, like *Deferribacteres*, there were no significant differences among the four groups.

**Fig 6:**
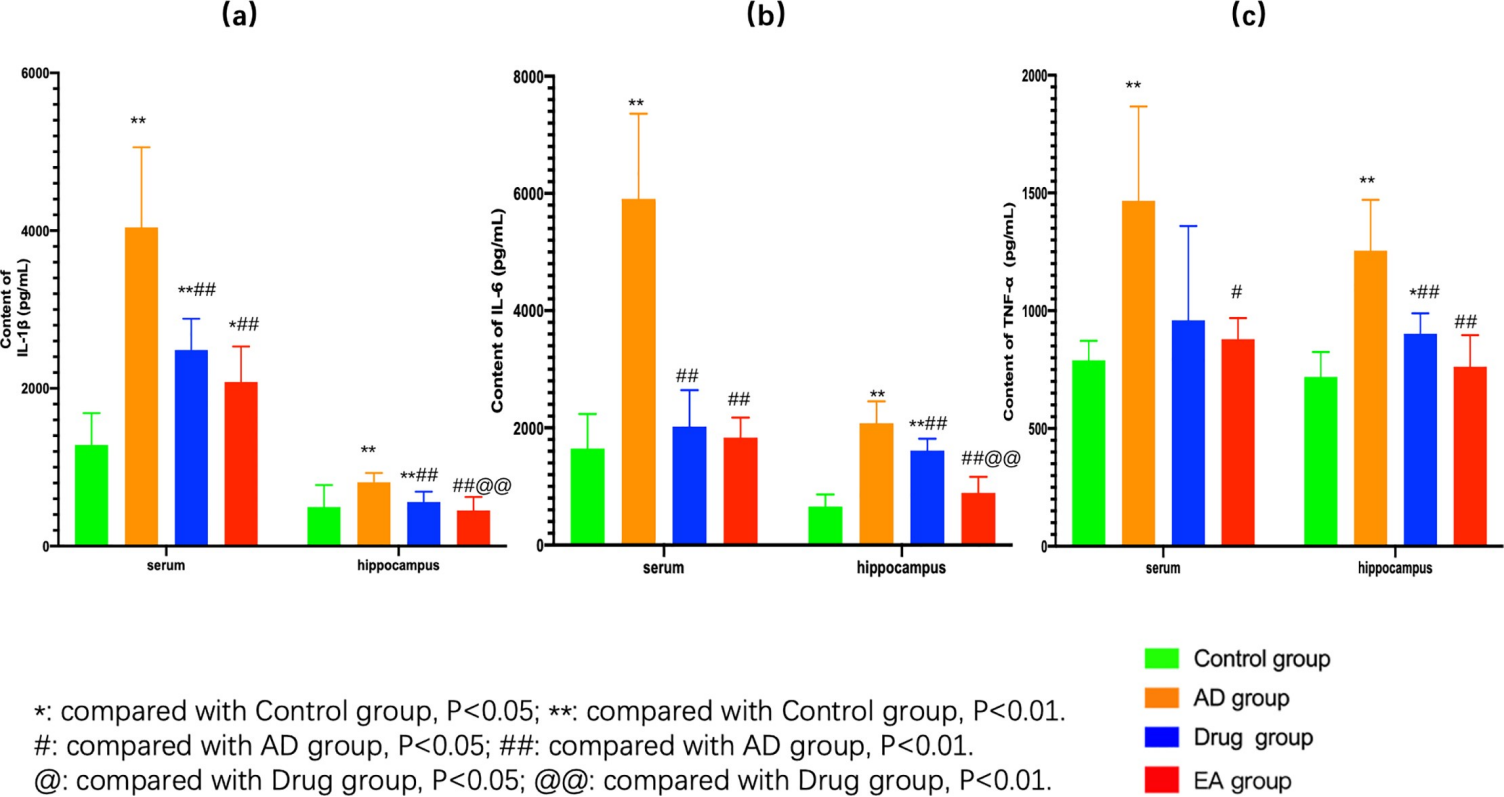
**(**a, comparation of IL-1β content in serum and hippocampus of each group; b, comparation of IL-6 content in serum and hippocampus of each group; c, comparation of TNF-α content in serum and hippocampus of each group.).

Third, we also wanted to explore the possible relationship between gut microbiota and the pathological process in the brain of AD animal model. Therefore, we used the ELISA to determine the relative proteins in serum and hippocampus, especially the pro-inflammation factors like IL-1β, IL-6 and TNF-α. As we could see from Fig 6, no matter in serum and hippocampus, compared with control group, the contents of IL-1β, IL-6 and TNF-α in the AD group were significantly higher (P<0.01). Generally, these factors in drug group and EA group were lower than AD group both in serum and hippocampus, especially for EA group. Compared to AD group, the content of each pro-inflammation factors (IL-1β, IL-6 and TNF-α) in EA group was significantly lower both in serum and hippocamps (the content of IL-1β and IL-6 in serum and hippocampus, p<0.01; the content of TNF-α in serum, p<0.05; the content of TNF-α in hippocampus, P<0,01). What’s more, compared with drug group, we could see the content of IL-1β and IL-6 in hippocampus were significantly lower (P<0.01). It was worth for further thinking that this change did not appear in the serum.

## 4 Discussion

Alzheimer’s disease is a neurodegenerative disease with insidious onset and progressive impairment of behavioral and cognitive functions including memory, comprehension, language, attention, reasoning, and judgement [28]. With the increasing rate of the incidence, mortality and disability year by year, it is concerned by the focus of the whole world and become one of the most threats in the field of world public health. Since the world health organization (WHO) published the report in 2012, “Dementia: a public health priority” more researchers and clinical doctors devoted themselves to find the pathological process and effective therapies of AD. In the latest decade, with the deepening of research and the accumulating of evidence, we have expanded our visons on the pathological characteristics and possible therapies of AD [29]. In current, scientists are gradually moving away from the simple assumption, as proposed in the original amyloid cascade hypothesis [2, 30] and Tau hyperphosphorylation hypothesis [31], to new theories of pathogenesis, including abnormal lipids transport [32–34], cerebrovascular dysfunction [35–37], and neuroinflammatory response caused by various chronic infections [38, 39].

Moreover, from the increasing evidence, we also found there might be the possible relationship between the microbiota inside human body and the process of AD [40, 41], especially the gut microbiota [42]. With the extensive studies on gut microbiota and the relationship between dysbacteriosis and some central nerve system diseases, the role of gut microbiota in the pathological process of AD should not be ignored. In the normal situation, the gut microbiota interacts with the host immune system and forms immune homeostasis by tolerating commensal antigens [43]. Therefore, gut microbiota could control the adaptive immune system [44]. In the pathological process of AD, we have found neuroinflammation and higher intestinal permeability appeared as the feature as the same time [45]. Besides, neuroinflammation factors, like IL-1β, IL-6 and TNF-α, could be observed not only in the brain but also in the serum of AD patients [46]. From this aspect, some researchers proposed that with the imbalance of gut microbiota and increasing of intestinal permeability, by releasing metabolites into blood, destroyed the blood-brain barrier (BBB) structure, and then affected and regulated the neuroinflammatory response in the brain of AD patients [45, 47–49]. Conversely, by restoring the balance of gut microbiota or increasing the number of beneficial microbiomes, we could control the neuroinflammatory reaction in AD brain at some extent, and then protect neurons and cognitive function [50]. This provided a therapeutic strategy and theoretical basis for some diet therapies and probiotics supplement therapy [17].

Both experimental and clinical studies found that the decrease of gut microbiota diversity was common in AD animal models and patients. In addition, the structure of gut microbiota has also changed [51]. In the animal model of intestinal malnutrition caused by ampicillin for one month, it was found that the spatial learning and memory ability of experimental animals was damaged and aggressive behaviors increased [52]. Then, the fecal samples of SAMP8 mice were analyzed, comparing with the SAMR1 mice, the gut microbiota diversity of SAMP8 mice was decreased, the *lactobacillius* was significantly reduced in the bacterial structure; Bacteroides, such as *lachnospiraceae_ NK4 A136*, *Alistites* and so on increased significantly [53, 54]. The clinical data of the study on gut microbiota of the elderly also confirmed the results of animal studies. The results showed that there were significant differences in the composition of intestinal flora between AD patients and healthy elderly in the level of species and species: the number of *Fiticutes* and *Actinobacteria* (especially *Bifidobacterum*) was significantly reduced, while the number of *Pseudobacteria* and *Proteobacteria* increased significantly [16].

In addition, with the decrease of the number of *Lactobacillus* and *Bifidobacteria* in the intestine of AD patients, the risk of intestinal inflammation, peripheral inflammation and neuroinflammation in brain is increased. The new research found that: the molecular model of Lactobacillus Enterobacter can effectively inhibit the pathogenic infiltration of peripheral immune cells into the central nervous system [55], thus reducing neuroinflammation [56]; After supplementation with *Lactobacillus* and Bifidobacterium, the cognitive function of AD model animals and mild cognitive impairment patients was improved and neuritis was significantly reduced [57, 58].

Nowadays, research on effects of microbiome with intervention of electroacupuncture were emerging. These studies focused on the mechanism of microbiome and immune regulation of electroacupuncture [43], such as electroacupuncture could decrease inflammation factors (lipopolysaccharide nterleukin-1beta and interleukin-6) via affecting the gut microbiome [59–61].

In this study, we intended to illustrated two points: 1) whether there were differences in gut microbiota between SAMP8 mice and SAMR1 mice, which were the common AD animal model in the research. 2) Whether the mechanism of improving cognitive function and protecting the neuron effect by electroacupuncture was related to the regulation of gut microbiota in AD animal model.

We organized the latest five years of original research from the angle of gut microbiota, which were all based on the AD animal model, what we found was interesting. Most of these studies used APP/PS1 transgenic mouse as the research subject [62–72], some studies applied 5xFAD mouse [73–76], there were only two studies collected the data of gut microbiota of SAMP8 mouse [77, 78]. As we known, SAMP8 mouse was used to represent sporadic Alzheimer’s disease (sAD), which account for over 95% of AD incidence rate [79]. Therefore, our study could be help for understanding the characteristics of SAMP8 mouse and accumulating evidence of gut microbiota of this mouse. From the results, we found there were significant differences between SAMP8 and SAMR1 mouse in the number of OTUs, the community structure and relative abundance of gut microbiota. Notably, in our study, we found the ratio of *Bacteroidia* and *Clostridia* was significantly different in SAMP8 and SAMR1 mouse, which were not mentioned in the two former studies. Studies have shown that the number of *Bifidobacterium*, *Lactobacillus* and *Bacteroidia* in the intestinal tract of the elderly with chronic diseases was gradually decreasing, while the number of *Clostridia* was gradually increasing [80, 81]. Our study was consistent with the above results, and provided evidence for further understanding of the characteristics of gut microbiota in AD model animals.

Our former studies proved some evidence of the improving cognitive function and protecting the neuron effect by electroacupuncture [23, 82–84]. These studies were mainly to explore the mechanism of electroacupuncture from the perspective of inhibiting neuroinflammatory response and regulating microglia activation [25, 26, 85]. In this study, we found electroacupuncture could affect the community structure of gut microbiota in SAMP8 mouse, especially up-regulating the ratio of *Bacteroidia* and *Clostridia*. Since we found the effects of electroacupuncture on gut microbiota, we wanted to find some evidence of relationship between these changes in gut to the brain. Thus, we determined the neuroinflammation factors IL-1β, IL-6 and TNF-α in the serum and in the hippocampus. As seeing from the results, IL-1β, IL-6 and TNF-α were both significantly increased in serum and hippocampus in SAMP8 mice, and electroacupuncture could down-regulate these inflammation factors. What should be noticed was the extent of the reduction of these factors in serum and hippocampus. The contents of IL-1β, IL-6 and TNF-α in the serum were higher than in hippocampus, especially IL-1β and IL-6. These might be one possible explanation of the mechanism of electroacupuncture: by balancing the gut microbiota, electroacupuncture could effectively inhibit the peripheral inflammatory response, and then played an effective role in controlling the neuroinflammatory response in hippocampus.

The potential biological mechanisms on how the electroacupuncture treatment could inhibit the peripheral and central nerve system inflammatory response by balancing the gut microbiota, including brain-gut microbiome-based neuroimmune [86, 87], RNA modification [88, 89], and genetic basis [90, 91]. There were three important aspects in this process. First, the impairment of intestinal barrier function was the primary trigger point. Intestinal barrier function played an important role between the body and gut microbiota, which was the basic premise of their symbiotic relationship [92]. The most direct consequence of intestinal barrier function damage caused by intestinal dysbacteriosis was to promote the "leakage" of a large number of proinflammatory metabolites of gut microbiota, such as lipopolysaccharide (LPS) [93]. Second, brain-gut axis was the core pathway of in this process. On the one hand, gut microbiome could regulate the vagus nerve via activation Toll-like receptor-4 on the surface of intestinal vagus nerve by LPS [94] as well as activation 5-hydroxytryptamine-3 (5-HT) receptor on the surface of intestinal vagus nerve by 5-HT [95], which could directly affect neuroinflammation in the brain. On the other hand, with the impairment of intestinal barrier function, the extravasation of gut microbiota metabolites would induce or exacerbate neuroinflammation in the brain through the circulatory system [96]. Third, activation of microglia in the brain caused by gut microbiota metabolites and relative inflammation factors finally accelerated the neuronal apoptosis in the process of AD [97]. In our current study, we already found the electroacupuncture could balance the gut microbiome in SAMP8 mice, however, we still need further studies to gain more evidence to explore and illustrate the mechanisms of electroacupuncture.

There are still some limitations in our study. First, limited numbers of SAMP8 mice and SAMR1 mice were used to generate the 16S rDNA amplicon sequencing data, which constrained the false discovery rate. Second, we only provided the evidence of electroacupuncture mechanism at the first aspect during the complex processes of AD pathology from the intestinal dysbacteriosis angle. Further research is needed. Third, even though we found the electroacupuncture treatment could inhibit the peripheral and central nerve system inflammatory response by balancing the gut microbiota, the potential causal effects have not been disclosed with enough biological experiments. A causal inference framework, like mendelian randomization analysis [98–100], or integrative network biology^15,103^, would help gain further causal effects of electroacupuncture treatment on improving the learning and memory abilities of Alzheimer’s disease through mediating the gut microbiota when combining the data on human gut microbiome and genome in Alzheimer’s disease.

## 5 Conclusion

Our current study identified the differences of gut microbiota in SAMP8 and SAMR1 mouse. In addition, we found the electroacupuncture treatment could inhibit the peripheral and central nerve system inflammatory response by balancing the gut microbiota. These provided more evidence for further understanding of the characteristics of gut microbiota in AD model animals and proposed one possible explanation of the mechanism of electroacupuncture, which could provide more evidence for helping AD patients.

## Supporting information

S1 Data (XLSX)
